# Supplementary material for: Molecular features of the UNC-45 chaperone critical for binding and folding muscle myosin
Source: Nat Commun. 2019 Oct 21;10:4781. doi: 10.1038/s41467-019-12667-8 (PMC6803673; doi:10.1038/s41467-019-12667-8)
Supplement: Supplementary file 1 — Supplementary Information [file 41467_2019_12667_MOESM1_ESM.pdf]

Supplementary Information

Molecular features of the UNC-45 chaperone critical for binding and folding muscle myosin

CONTENT

Supplementary Figures 1-6, Supplementary Tables 1-4

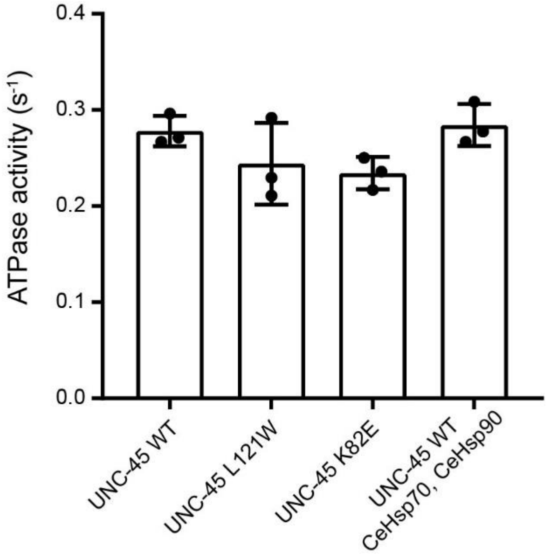

Supplementary Figure 1. Actin-activated ATPase activity determined for the monomeric MHC-B peak after SEC from the indicated co-expression studies (n = 3, data represent mean ± s.d.)

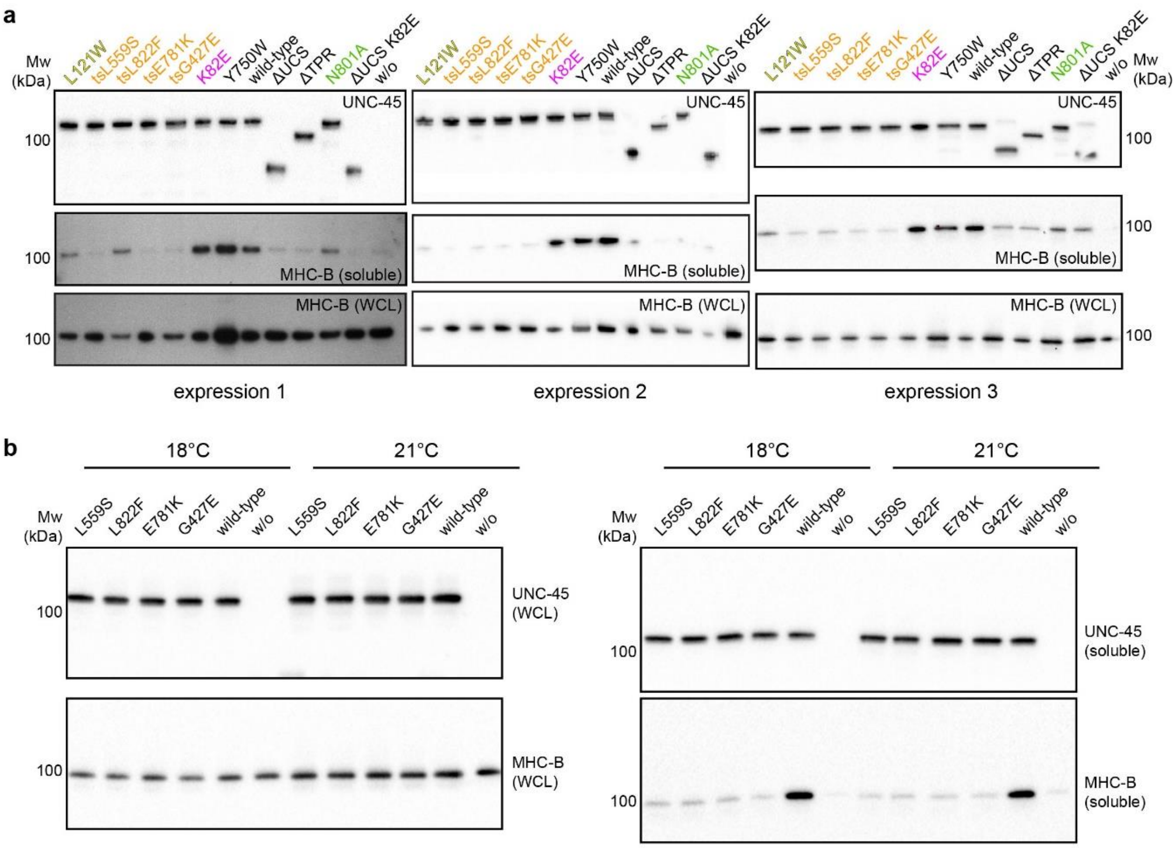

Supplementary Figure 2. Co-expression of MHC-B with UNC-45 mutant proteins. (**a**) Western blot analysis of co-expression of MHC-B with indicated UNC-45 mutant proteins in insect cells. Whole cell lysates (WCL) and soluble fractions are shown for MHC-B. Input was normalized to soluble UNC-45 levels (top panels). (**b**) Comparison of co-expression of MHC-B with UNC-45 *ts*-mutants at 18°C (permissive temperature for *ts*-worm development) and 21°C (standard temperature for insect cell expression in this study). Western blots show whole cell lysates (WCL) and soluble fractions for the UNC-45 proteins and MHC-B.

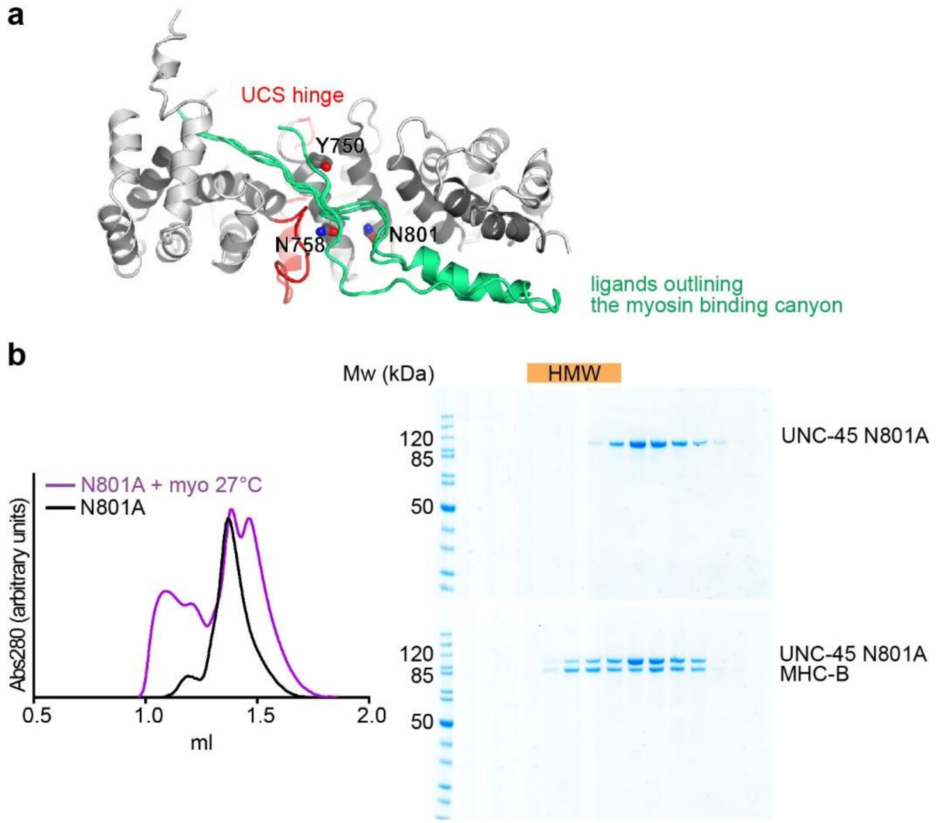

Supplementary Figure 3. Analysis of UCS domain to identify myosin folding mutants. (**a**) Ligands co-crystallized with the ARM repeat domain of β-catenin (green) mapped onto the UCS domain of wild-type UNC-45 (grey, PDB code: 4i2z). Residues at the center of the outlined myosin binding canyon (N758 and N801) or bordering the canyon (Y750) are highlighted. (**b**) Analytical SEC and SDS-PAGE analysis of UNC-45 N801A incubated with and without myosin at 27°C. High molecular weight fractions (HMW) are indicated.

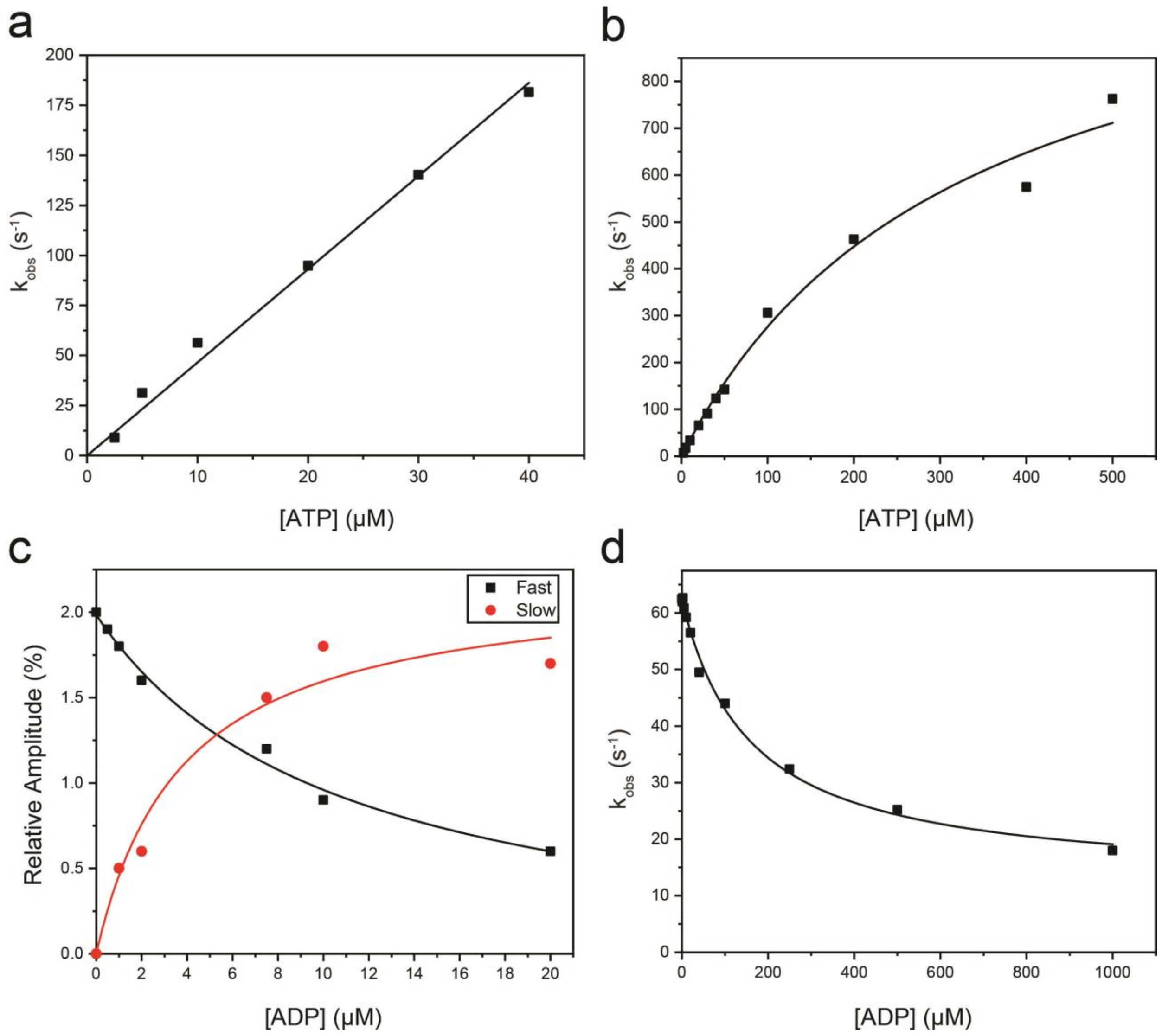

Supplementary Figure 4. Biochemical characterization of MHC-B by stopped-flow analysis. The average of two independent measurements are given in Supplementary Table 1. (**a**) ATP binding to MHC-B. The effect of ATP concentration on k_{obs} for ATP-induced dissociation of pyrene-actin:MHC-B. The gradient generates second order rate constant of ATP binding (K_1k_{+2}) value of 6.1 $\mu M^{-1}s^{-1}$. (**b**) The effect of ATP concentration on k_{obs} for ATP-induced dissociation of pyrene-actin:MHC-B. The gradient generates a second order rate constant of ATP binding, $K'_1k'_{+2}$, value of 3.6 $\mu M^{-1}s^{-1}$. The affinity of MHC-B for ATP, $1/K'_1$, is 340 µM and the maximum rate of ATP-induced dissociation, k'_{+2}, is 1198 s^{-1}. (**c**) Affinity of MHC-B for ADP. The relative amplitudes of the fast and slow phases plotted against ADP concentration, which has a hyperbolic dependence and generates an ADP affinity of 7.7 µM. (**d**) ADP affinity for actin:MHC-B. k_{obs} plotted as a function of ADP concentration fitted with a hyperbolic equation resulting in an ADP affinity ($K'_6K'_7$) value of 143 µM.

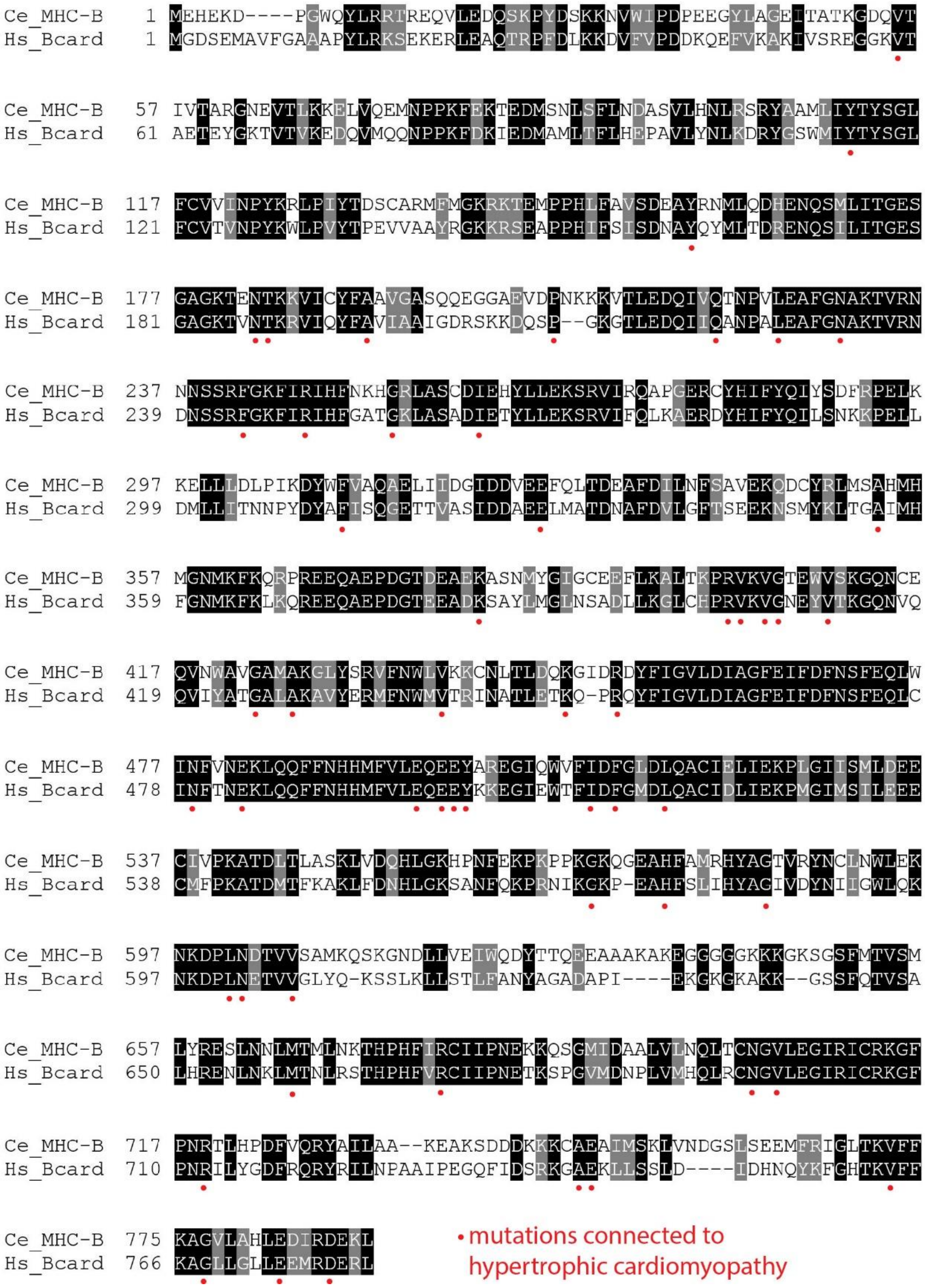

Supplementary Figure 5. Sequence alignment of MHC-B and human β-cardiac myosin motor domains. Identical residues are highlighted in black, similar residues in grey. Mutations connected with hypertrophic cardiomyopathy are indicated by a red dot.

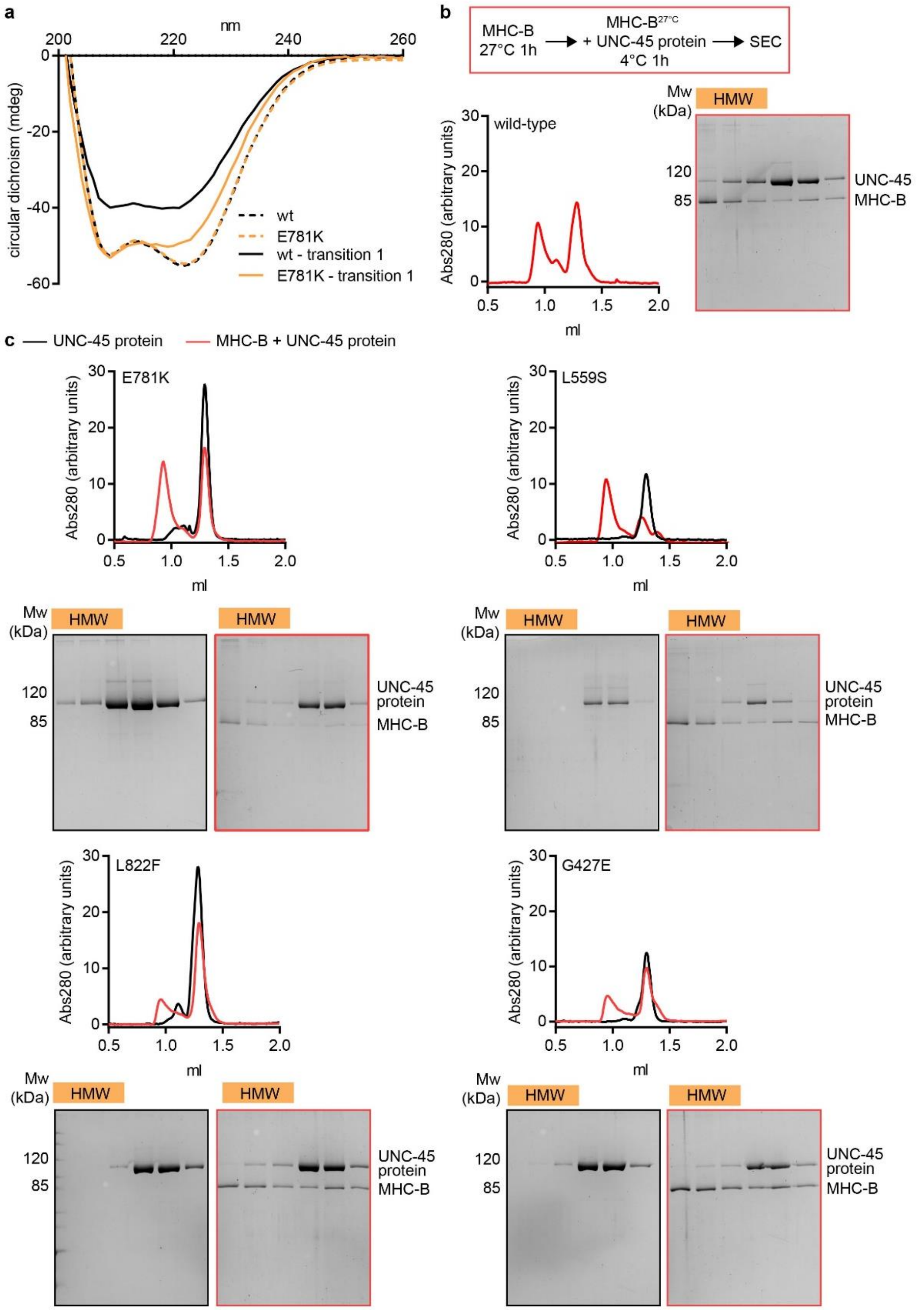

Supplementary Figure 6. Analysis of UNC-45 *ts*-mutants in *in vitro* assays. (**a**) CD spectra for wild-type UNC-45 and E781K. (**b**) Experimental outline illustrating the sequence of events: MHC-B was first incubated at 27°C for 60 min to induce protein unfolding. Following this step UNC-45 and MHC-B were incubated together at 4°C for 60 min and analyzed by SEC. UNC-45 which interacts with unfolded MHC-B is present in the high molecular weight (HMW) fractions. (**c**) Same analysis as in (b) for the indicated UNC-45 *ts*-mutants.

Supplementary Table 1: Kinetic properties of MHC-B compared to human β-cardiac S1. Data represent mean ± s.e.m. Raw data and calculations are shown in Supplementary Figure 4. Data are shown for the constants depicted for the actin-myosin (a) and myosin (b) ATPase cycle. M = myosin; A= actin; T = ATP; D = ADP; P = P_i. Dashed (-) interactions represent a weakly bound complex, and dotted (·) interactions represent a strongly bound state. An equilibrium constant, K_i can be defined as k_{+i}/k_{i}.

a

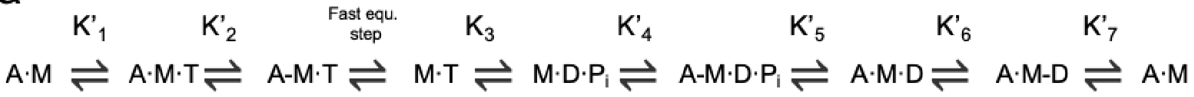

b

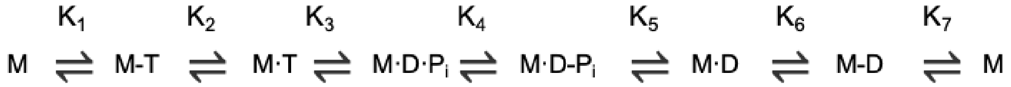

	C. elegans MHC-B	human β-cardiac S1
ATP binding to myosin		
K_1k_{+2} ($\mu M^{-1}s^{-1}$)	6.8 ± 1.2	5.8 ± 0.2
ADP binding to myosin		
K_6K_7 (μM)	7.55 ± 0.5	0.53 ± 0.06
k_{+6} (s^{-1})	12.7 ± 0.7	0.63 ± 0.03
ATP binding to acto.myosin		
$K'_1k'_{+2}$ ($\mu M^{-1}s^{-1}$) at 20°C	3.6 ± 0.4	4.4 ± 0.3
$1/K'_1$ (μM)	324.2 ± 67.9	327.9 ± 53.3
k'_{+2} (s^{-1})	1172.8 ± 125.6	1543 ± 100
ADP affinity for acto.myosin		
$K'_6K'_7$ (μM)	131.5± 19.3	6.1 ± 0.7
k'_{+6} (s^{-1})	189 ± 42.5	58.7 ± 3.3
S1 affinity for actin		
K_A (nM)	10.3 ± 3.7	10 ± 1.8

Supplementary Table 2: **Data collection and refinement statistics.**

	MHC-B	Unc-45 G427E	Unc-45 L822F	Unc-45 ΔTPR
PDB code	6QDJ	6QDK	6QDL	6QDM
**Data collection**				
Space group	$P2_1$	$P6_122$	$P2_1$	$C2$
Cell dimensions				
a, *b*, *c* (Å)	54.11, 111.96, 84.74	86.91, 86.91, 718.54	54.12, 114.32, 85.01	157.39, 97.61, 148.88
α, β, γ (°)	90, 97.44, 90	90, 90, 120	90, 107.99, 90	90, 93.41, 90
Resolution (Å)	85-1.9 (2.05-1.90) *	30-3.4 (3.49-3.4)	47-2.9 (3.09-2.9)	49-3.8 (4.01-3.80)
R_{meas} (%)	4.5(45.4)	12.0 (84.8)	11.9 (67.5)	6.2 (127.5)
$I / \sigma I$	19.6(3.1)	12.0 (1.9)	8.5 (1.4)	9.1 (1.1)
Completeness (%)	93.8(78.0)	99.6 (96.2)	97.0 (82.3)	98.9 (96.2)
Redundancy	3.3(2.9)	18.2 (8.8)	3.1 (2.7)	3.3 (3.1)
**Refinement**				
Resolution (Å)	85-1.9	30-3.4	47-2.9	49-3.8
No. reflections	76,202	23,652	20,543	22,138
R_{work} / R_{free}	18.5/21.3	28.7/32.0	22.1/26.8	28.0/31.0
No. atoms				
Protein	6,130	6,851	6,931	11,815
Ligand/ion	131	-	-	-
Water	237	-	-	-
B-factors				
Protein	38.6	96.5	63.5	200
Ligand/ion	45.5	-	-	-
Water	40.6	-	-	-
R.m.s. deviations				
Bond lengths (Å)	0.012	0.008	0.007	0.013
Bond angles (°)	1.32	1.07	0.99	1.48

*Number of xtals: 1. *Values in parentheses are for highest-resolution shell.

Supplementary Table 3: **Structural homologs of indicated UCS domains identified by a DALI search.**

	wt, extended (4i2z)		wt, curved (5mzu)		ΔTPR, molecule B		tsL822F		tsG427E	
**rank**	**structural homolog**	**Z-score**	**structural homolog**	**Z-score**	**structural homolog**	**Z-score**	**structural homolog**	**Z-score**	**structural homolog**	**Z-score**
**UCS proteins**										
	DmUNC-45	35.3	DmUNC-45	26.5	DmUNC-45	31.6	DmUNC-45	36.9	DmUNC-45	29.6
	She4p	28.1	CeUNC-45, extended	23.1	She4p	25.4	CeUNC-45, extended	31.3	She4p	24.0
	CeUNC-45, curved	17.0	She4p	22.2	CeUNC-45, extended	24.8	She4p	28.8	CeUNC-45, extended	23.8
					CeUNC-45, curved	19.4	CeUNC-45, curved	16.0	CeUNC-45, curved	16.4
**Non UCS proteins**										
**1**	β-catenin	24.2	catenin delta-1	19.3	β-catenin	20.9	importin α	24.9	vacuolar protein 8	20.4
**2**	engineered protein OR497	24.0	β-catenin	18.9	importin α	20.5	engineered protein or497	24.7	importin α	20.1
**3**	importin α	23.9	importin α	18.6	vacuolar protein 8	19.9	xpg2 peptide	24.7	GTP-binding nuclear protein ran	19.8
**4**	vacuolar protein 8	23.8	GEF srm1	17.8	GEF srm1	19.8	karyopherin α	24.4	GEF srm1	19.8
**5**	GEF srm1	23.8	engineered protein OR497	17.1	karyopherin α	19.5	GEF srm1	24.3	β-catenin	19.6
**6**	karyopherin α	23.4	plakoglobin	17.1	protein humpback-2	19.0	β-catenin	24.2	karyopherin α	19.6
**7**	GTP-binding nuclear protein ran	22.8	RAP1 GTPase-GDP dissociation stimulator 1	16.4	plakoglobin	18.0	vacuolar protein 8	23.9	protein humpback-2	19.1
**8**	nuclear cap-binding protein subunit 1	22.7	adenomatous polyposis coli protein	16.3	APC variant protein	17.8	flap endonuclease 1 (fen1) peptide	23.8	plakoglobin	18.1

Supplementary Table 4: **Primers used in this study.**

Primers used for site-directed mutagenesis of UNC-45 constructs in pET21a	
tsUNC-45 G427E	ATTACAATGCTTCAAGAACCAGTTGATATTGGA
tsUNC-45 L559S	CTCTCCTATTTGTCTTCGGATGCTGATGTTAAG
tsUNC-45 E781K	ATTCCAAAGATTGAGAAATTCTGGTTTATGACG
tsUNC-45L822F	ACCGATCGTCTGAAATTCTGGGTTCTCTACTCG
UNC-45 N801A	Forward:<br>CGCTGAGCTTCTTCTCGCGTTGCTCTTTTTCGAGA<br>Reverse:<br>TCTCGAAAAAGAGCAACGCGAGAAGAAGCTCAGCG

Primers used for generating expression constructs for insect cells		
	Forward	Reverse
MHC-B in pFastBacDual	GATCGAATTCATGGAGCACGAGAAGGAC	CATGTCTAGACTAGTGATGGTGGTGATGGTGGAGCTTCTCGTCACGGAT
NMY-2 in pFastBacDual	GATCGCGCGCATGACATCATCTCGACAA	CATGTCTAGACTAGTGATGGTGGTGATGGTGAAGCTTCTGATCACGCAT
UNC-45 in pFastBacDual	GATGCCCGGGATGGTTGCTCGAGTACAG	together with MHC-B:<br>GATGGCATGCTTATTTTTCGAACTGCGGGTGGCTCCATTCCTGAATGGTGCTCAT
		together with NMY-2:<br>GATGCCATGGTTATTTTTCGAACTGCGGGTGGCTCCATTCCTGAATGGTGCTCAT
Hsp70 in pFastBacDual	GATGCCCGGGATGGACTACAAAGACGATGACGACAAGAGTAAGCATAACGCTGTT	GATCGCTAGCTTAGTCGACCTCCTCGAT
Hsp90 in pFastBacDual	GATGCCCGGGATGGACTACAAAGACGATGACGACAAGTCCGAGAACGCCGAAACC	GATCGCTAGCTTAGTCGACCTCCTCCAT
MHC-B in pACEBac1	CGGATCCCGGTCCGAAACCATGGAGCACGAGAAGGACCCAG	CCCCAGAACATCAGGTTAATGGCGCTAATGATGGTGGTGATGGTGGAGC
UNC-45 full-length proteins in pIDC derivative	CTGGAAGTTCTGTTCCAGGGGCCCATGGTTGCTCGAGTACAGACTGC	CCCCAGAACATCAGGTTAATGGCGTTATTCCTGAATGGTGCTCATTTGATTTTCG
UNC-45 ΔUCS in pIDC derivative	CTGGAAGTTCTGTTCCAGGGGCCCATGGTTGCTCGAGTACAGACTGC	CCCCAGAACATCAGGTTAATGGCGTTATTTCATCGTTGCTTTCGAAATGTCGTC
UNC-45 ΔTPR in pIDC derivative	CTGGAAGTTCTGTTCCAGGGGCCCACCACTTCACTGGCTAATAAGGTAACTG	CCCCAGAACATCAGGTTAATGGCGTTATTCCTGAATGGTGCTCATTTGATTTTCG
Hsp90 in pACEBac1 derivative	CGGATCCCGGTCCGAAACCATGGACTACAAAGACGATGACGACAAG	CCCCAGAACATCAGGTTAATGGCGTTAGTCGACCTCCTCCATGCG
Hsp70 in pACEBac1 derivative	CCCATACGATGTTCCAGATTACGCTAGTAAGCATAACGCTGTTGGAATCG	CCCCAGAACATCAGGTTAATGGCGTTAGTCGACCTCCTCGATCGTTG
MHC-B in pACEBac1 derivative	GATCCCGGTCCGAAACCATGGAGCACGAGAAGGACCCAG	CCCCAGAACATCAGGTTAATGGCGCTAATGATGGTGGTGATGGTGGAGC
